# Challenges of genetic diagnosis of inborn errors of metabolism in a major tertiary care center in Lebanon

**DOI:** 10.3389/fgene.2022.1029947

**Published:** 2022-11-18

**Authors:** Doaa O. Salman, Rami Mahfouz, Elio R. Bitar, Jinane Samaha, Pascale E. Karam

**Affiliations:** ^1^ Department of Pediatrics and Adolescent Medicine, American University of Beirut, Beirut, Lebanon; ^2^ Department of Pathology and Laboratory Medicine, Faculty of Medicine, American University of Beirut, Beirut, Lebanon; ^3^ Faculty of Medicine, American University of Beirut, Beirut, Lebanon; ^4^ Inherited Metabolic Diseases Program, American University of Beirut Medical Center, Beirut, Lebanon

**Keywords:** inborn errors of metabolism (IEM), next gen sequencing, whole exome sequencing, Arab countries, diagnostic yield

## Abstract

**Background:** Inborn errors of metabolism are rare genetic disorders; however, these are prevalent in countries with high consanguinity rates, like Lebanon. Patients are suspected, based on a combination of clinical and biochemical features; however, the final confirmation relies on genetic testing. Using next generation sequencing, as a new genetic investigational tool, carries several challenges for the physician, the geneticist, and the families.

**Methods:** In this retrospective study, we analyzed the clinical, biochemical, and genetic profile of inborn errors of metabolism suspected patients, seen at a major tertiary care center in Lebanon, between 2015 and 2018. Genetic testing was performed using next generation sequencing. Genotype-phenotype correlation and diagnostic yield of each testing modality were studied.

**Results:** Out of 211 patients genetically tested, 126 were suspected to have an inborn error of metabolism. The diagnostic yield of next generation sequencing reached 64.3%. Single gene testing was requested in 53%, whole exome sequencing in 36% and gene panels in 10%. Aminoacid disorders were mostly diagnosed followed by storage disorders, organic acidemias and mitochondrial diseases. Targeted testing was performed in 77% of aminoacid and organic acid disorders and half of suspected storage disorders. Single gene sequencing was positive in 75%, whereas whole exome sequencing diagnostic yield for complex cases, like mitochondrial disorders, reached 49%. Good clinical and biochemical correlation allowed the interpretation of variants of unknown significance and negative mutations as well as therapeutic management of most patients.

**Conclusion:** Tailoring the choice of test modality, by next generation sequencing, to the category of suspected inborn errors of metabolism may lead to rapid diagnosis, shortcutting the cost of repeated testing. Whole exome sequencing as a first-tier investigation may be considered mainly for suspected mitochondrial diseases, whereas targeted sequencing can be offered upon suspicion of a specific enzyme deficiency. Timing and modality of gene test remain challenging, in view of the cost incurred by families.

## Introduction

Inborn errors of metabolism (IEM) are rare disorders, but collectively their incidence may reach worldwide 1 in 1900 births (Tebani et al., 2016). The role of consanguinity in the prevalence of these genetic disorders is well established. In most Arab communities, consanguineous marriage is still a tradition, and intrafamilial unions account for 20%–50% of all marriages (Charng et al., 2016). In Lebanon, the overall rate of consanguinity reaches up to 35.5% (Barbour and Salameh, 2009) with 67% of the reported genetic diseases following an autosomal recessive inheritance pattern (Nakouzi et al., 2015). In parallel, the incidence of IEM detected by neonatal screening in Lebanon is estimated at 1 in 1,482 (Khneisser et al., 2015), compared to 1 in 2,760 births in developed countries, like in Spain (Martin-Rivada et al., 2022). Neonatal screening is not systematic yet in Lebanon, therefore patients with IEM may represent a diagnostic challenge for physicians. These inherited disorders may occur at any age, from the neonatal period to adulthood, with various non-specific clinical presentations. Thorough clinical assessment, comprehensive family history along with biochemical investigations are key for diagnostic orientation; however, the final diagnostic confirmation relies on genetic testing. Since 2006, the emerging Next Generation Sequencing (NGS), including single gene, multi-gene panel, and whole exome/genome sequencing (WES/WGS) became an attractive diagnostic tool, compared to traditional sequencing methods (Neveling et al., 2013; LaDuca et al., 2017). However, the major challenges reside in the interpretation of NGS results. Variants of uncertain significance (VUS), and novel mutations may be difficult to ascertain as disease-causing; from a clinical standpoint, the physician would be faced with the dilemma of sharing an unsure diagnosis with the patients, adding to their anxiety (Claustres et al., 2014), while being unable to offer a specific management. Nevertheless, good clinical and biochemical correlation may allow the clinician to consider VUS as relevant and related to the observed phenotype. Negative results are even more stressing for both the physician and the patient, especially when all clinical and biochemical clues along with a positive family history suggest an IEM, and yet no mutation is found by NGS.

On another note, NGS use as routine investigational tool in daily practice can represent a real challenge in countries where genetic testing is not covered by any third-party payer.

Scarce studies on NGS utility in the diagnosis of genetic disorders are available from Arab countries, featuring mostly neurogenetic disorders and syndromes with few IEM cases reported (Yavarna et al., 2015; Al-Shamsi et al., 2016; Alfares et al., 2017; Megarbane., 2018). The adoption of NGS techniques in Lebanon during the last decade enhanced the diagnosis of various genetic disorders including IEM; however, the use of these advanced genetic tests slowed down with the Lebanese economic crisis as of 2019 (Bizzari et al., 2021). We report in this retrospective review the challenges of genetic diagnosis of IEM at a major tertiary care center in Lebanon.

## Materials and methods

We performed a retrospective review of charts of patients referred to the Inherited Metabolic Diseases Program at the American University of Beirut Medical Center (AUBMC) between 2015 and 2018, who underwent NGS diagnosis. Age at diagnosis, family history, consanguinity, clinical data, biochemical work-up, and genetic tests were recorded. Suspected amino acid and organic acid disorders were investigated by high performance liquid chromatography (HPLC) for amino acids, gas chromatography mass spectrometry (GC/MS) for organic acids and mass spectrometry (MS/MS) for acylcarnitine and amino acids. Enzyme assays on dried blood spots for lysosomal disorders, galactosemia and biotinidase deficiency as well as urine glycosaminoglycans for mucopolysaccharidosis were sent to accredited reference laboratories outside AUBMC. Gene studies were done, depending on availability, at the Molecular unit at AUBMC or referred to outside genetic centers, mainly to Germany (Centogene AG). Informed consent was obtained for all patients. Requested genetic tests according to each case ranged from single gene sequencing to multigene panels, whole exome and/or whole genome sequencing. Sanger confirmation was done for pathogenic/likely pathogenic variant or variant of uncertain significance (VUS). All reports were approved and released by certified geneticists. Variants were reported by the testing laboratory and classified based on each laboratory’s own database. All reported variants were classified as pathogenic (class 1) or likely pathogenic (class 2), of uncertain significance (class 3), likely benign (class 4), benign (class 5) or disease-associated variant (class 6) according to the classification criteria of the American College of Medical Genetics and Genomics. Diagnostic yield of each genetic test modality (single gene sequencing, panel or WES) was calculated by dividing the number of cases identified with class 1 or class 2 mutations over the total number of patients who underwent the genetic test. Variant novelty was assessed using public variant repositories, including Gene Card, a validated database containing all mentioned variants in ClinVar and Humsavar, and reported variants in the literature. This study was approved by the Institutional Review Board at the American University of Beirut under protocol number BIO 2018-0381.

### Statistical analysis

Data was analyzed using IBM Corp. Released 2012. IBM SPSS Statistics for Windows, Version 21.0. Armonk, NY: IBM Corp.

## Results

### Demographic data

A total of 211 patients, seen at the Inherited Metabolic Diseases Program between 2015 and 2018, underwent genetic testing for diagnostic purposes. Among these, 81 cases had non- specific symptoms (neurological, cardiac and/or failure to thrive) with negative metabolic work-up and they were finally diagnosed with various non-IEM genetic disorders.

Data of the remaining 126 patients, 66 males (52%) and 60 females (48%), suspected to have an IEM with suggestive clinical presentation and/or positive biochemical profile, was analyzed. Age at time of evaluation by genetic testing ranged from the prenatal period to 42 years, with 66% below 5 years and 4% above 18 years. Consanguinity was positive in 67% of cases and family history of affected siblings was recorded in 53%. Genetic testing identified mutations in 103 patients, while the remaining 23 had negative results.

### Clinical presentation

**FIGURE 1:**
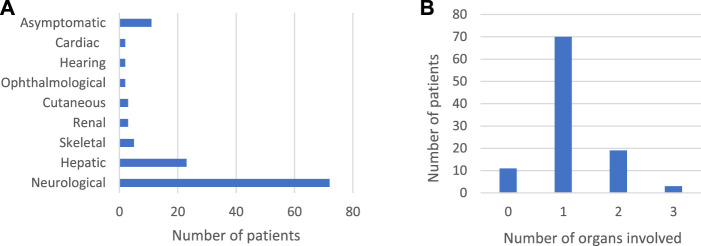
Clinical characteristics of 103 IEM patients confirmed by genetic testing. **(A)**: Systems involved in 103 IEM patients **(B)**: Number of organs involved.

The clinical characteristics of the 103 IEM patients were categorized according to the system involved. Most of the patients had neurological, and to a lesser extent, hepatic presentation (Figure 1). Around 11% were genetically tested although they were asymptomatic, as they had positive neonatal screening confirmed by metabolic work-up (7%) or a positive family history of affected siblings (4%).

### Molecular characteristics of inborn errors of metabolism categories

In this cohort of 103 patients with identified mutations, a total of 80 cases (78%) had pathogenic variants (62%), or likely pathogenic variants (16%) (Supplementary Table S1). VUS were detected in 23 patients (22%) (Table 1) while novel mutations were detected in 31 patients out of 103 (30%) (Table 2). Both VUS and novel mutations were mostly found in mitochondrial diseases, followed by aminoacid and organic acid disorders. Most of the VUS (79%) and all novel mutations were highly correlated with the clinical and biochemical profile of the probands and were considered clinically valid. Almost half of the VUS variants were missense mutations; the effect of the remaining variants was unknown.

**TABLE 1:** Variants of unknown significance identified in 23 IEM patients with phenotype correlation.

Gene	Variants	Effect	Type	Inheritance mode	Disease	Age^b^	Clinical phenotype	Biochemical phenotype
*PAH*	c.969 + 5G>A	NA	Intron	AR	PKU	8m	Neuro	HPA, normal biopterin
*PAH*	c.898G>T; c.1066-11G>A IVS10-11G>A	p.Ala300Ser	Missense; intron	AR	PKU mild	11y	NBS-as	HPA, normal biopterin
*PTS*	c.373G>A^a^	p.Gly125Arg^a^	Unknown	AR	BH4 type A	5y	Neuro	HPA
*BCKDHB*	c.995C>T	p.Pro332Leu	Missense	AR	MSUD	1y	Neuro	BCAA, allo ile increased
*TAT*	c.1262C>G^a^	p.Thr421Arg^a^	Unknown	AR	TYR-II	1y	NBS-as	Tyr increased, SA negative
*HCFC1*	c.1355C>T	p.Ala452Val	Missense	XLR	Cbl X	20m	Neuro	Organic aciduria, hcy
*GCDH* ^c^	c.349G>A	p.Gly117Arg	Missense	AR	GA type I	8y	Neuro	Organic aciduria
*GCDH* ^c^	c.349G>A	p.Gly117Arg	Missense	AR	GA type I	12y4m	Neuro	Organic aciduria
*TIMM50*	c.1114G>A^a^	p. Gly372Ser^a^	Unknown	AR	3-MGC type IX	3y	Neuro	Organic aciduria
*COX5B AARS2*	1)COX5B (c.374A>G)	1) p.Q125R	1) Unknown	AR	1)Cyt c oxidase	2y10m	Neuro	Lactic acidosis hyperlalaninemia
	2)AARS2	2)	2)		2)Oxphos type 8			
	A](c.1398C>T);	A] p.S466S	A] Synonymous					
	B] (c.1162C>T)	B] p. P388S	B] missense					
*EARS2*	c.209G>T	p.Ser70IIe	Missense	AR	COXPD12	1y	Neuro	Lactic acidosis, hyperlaninemia
*SDHA*	c.1816T>C; c.1799G>A	p.Tyr606His; p.Arg600Gln	Missense	AR/AD	Leigh	2y	Neuro	Lactic acidosis, hyperlaninemia
*1)NDUFA11*	1)c.614C>G	1) p.Ala205Gly	1) Missense	AR/AD	Leigh	1y	Neuro	Lactic acidosis, hyperlaninemia
*2)DNM1L*	2) c.1470A>G	2) p.Glu490Glu	2) Synonymous					
*3)PDHB*	3) c.621G>A	3) p.Gly207Gly	3) Synonymous					
*NDUFS1*	c.634A>C^a^	p.Thr212Pro^a^	Unknown	AR	Leigh	8m	Neuro	Lactic acidosis, hyperlaninemia
*MECR*	c.A517G^a^	p.R173G^a^	Unknown	AR	DYTOABG	6y	Neuro ophtalmo	Normal
*HADHA*	c.955G>A	p.Gly319Ser	Missense	AR	TFP	2y	Neuro	Dicarboxylic aciduria, CPK increase
*1)GLB1*	1) c.8G>T	1) p.Gly3Val	Unknown	AR	GM1 Gangliosidosis	30d	Neuro	Mildly increased CPK
*2) POLR3A*	2) c.755T>C	2) p.Leu252Pro						
*PEX3* ^b^	c.454A>C	P.Thr152Pro	Unknown	AR	Peroxisome biogenesis 10A	1y7m	Neuro	Increased phytanic acid
*SLC37A4*	c.1348G>A	NA	Unknown	AR	GSD Ib	10m	Liver	Hypoglycemia, neutropenia, transaminitis
*PHKA2*	c.3336 + 5G>A	NA	Unknown	XLR	GSD Type IXa1/IXa2	3y	Neuro	Hyperphenylalaninemia
*SLC52A1*	c.1292del	p.Ser431Thrfs^b^ 13	Unknown	AD	Riboflavin deficiency	5y	Kidney	Metabolic acidosis
*GALT*	c.707G>T	p.Ser236Ile	Unknown	AR	Galactosemia	3y	Neuro	GALT deficiency
*ALG12*	c.535G>A	p.Ala179Thr	Missense	AR	CDG type 1	5m	Neuro	Transferrin electrophoresis abn

^a^ Unreported.

^b^ At genetic diagnosis.

^c^ Siblings.

AR, autosomal recessive; XL, X-linked; AD, autosomal dominant; m, month; y, year; d, day; NBS, newborn screening; as, asymptomatic; Allo ile, alloisoleucine; HCY, homocystinuria; PKU, phenylketonuria; HPA, hyperphenylalaninemia; BH4, tetrahydrobiopterin; MSUD, maple syrup urine disease; BCAA, branched-chain amino acids; allo-ile, allo-isoleucine; TYR, tyrosinemia; Cbl, Cobalamin; hcy, homocystinuria; GA, glutaric acidemia; 3-MGC type IX, 3-Methylglutaconic aciduria type IX; cyt c oxidase, cytochrome c oxidase; Oxphos, oxidative phosphorylation; COXPD12, oxidative phosphorylation deficiency type 12; DYTOABG, dystonia with optic atrophy and basal ganglia abnormalities; TFP, trifunctional protein deficiency; CPK, creatine phosphokinase; GSD, glycogen storage disease; GALT, galactose-1-phosphate uridyl transferase; CDG, congenital disorders of glycosylation.

**TABLE 2:** Novel mutations detected in 31 patients clinically and biochemically suspected of IEM.

Disorders	Gene (OMIM #)	Molecular genetics	Protein effect	Inheritance mode	Class^a^	Disease	Age^b^	Clinical phenotype
Amino acids	*PTS (612719)*	c.373G>A	p.Gly125Arg	AR	Class 3	BH4 type A	5y	Neuro
	*QDPR (612676)*	c.197A>G	p.Gln66Arg	AR	Class 1	BH4 type C	22m	Neuro
	1.*FAH (613871*	c.1054A>G	NA	AR	Class 1	TYR-I	2y	Liver kidney
	2.*TAT 613018)*	c.1262C>G	p.Thr421Arg	AR	Class 3	TYR-II	1y	NBS-as
	3.*OCA2 (611409)*	c.2036G>A	p.Trp679^b^	AR	Class 2	Albinism -II	5m	Skin
	4.*DBT (248610)*	c.224G>A (exon 9)	p.Gly75G	AR	Class 1	MSUD	4y	Neuro
Organic acids	*ASPA (608034)*	c.497C>T	p.Thr166IIe	AR	Class 6	Canavan	2y	Neuro
	5.*HMGCL (613898)*	c.918dup	p.Cys307Leufs^b^9	AR	Class 2	HMG CoA lyase	1m	Neuro
	6. *TIMM50 (607381)*	c.1114G>A	p. Gly372Ser	AR	Class 3	3-MGC type IX	3y	Neuro
Mitochondrial	*ISCA2(615317)*	c.37_39del; c.190_192del	p.Thr13del; p.Thr64del	AR	Class 2	MMDS4	10m	Neuro
	*QRSL1 (617209)*	c.173G>T	p.Arg58IIe	AR	Class 2	COXPD40	1m	Cardiac
	*FBXL4 (605654)*	c.605C>G	p.Thr202 Arg	AR	Class 2	MTDPS	13y	Neuro
	*COX5B (123866)*	c.374A>G	p.Q125R	AR	Class 3	1)Cyt c oxidase 2)Oxphos type 8	2y10m	Neuro
	*SURF1 (185620)*	c.870delT	p.F290fs	AR	Class 1	Leigh	1y9m	Neuro
		c.370G>A	p.Gly124Arg	AR	Class 1	Leigh	1y	Neuro
	*NDUFS1 (157655)*	c.634A>C	p.Thr212Pro	AR	Class 3	Leigh	8m	Neuro
	*MPV17(137960)*	c.284dup	p.Phe96Leufs^b^17	AR	Class 2	MTDPS	1y	Liver
Storage	*SMPD1 (607608)*	c.923del	p.Phe308Serfs^b^77	AR	Class 1	Niemann-pick type A/B	6m	Neuro
	7.*GLB1 (611458)*	c.320G>A	p.Arg107His	AR	Class 1	GM1gangliosidosis	1y	Neuro
	8.*POLR3A (614258)*	c.755T>C	p.Leu252Pro	AR	Class 3	GM1gangliosidosis	3d	Neuro
	9.*ARSB (611542)*	c.691-13A>G	NA	AR	Class 6	MPS type VI	14y	Skeletal, cardiac
	10.*PEX3(603164)*	c.454A>C	P.Thr152Pro	AR	Class 3	Peroxisome biogenesis 10A	1y7m	Neuro
	11.*AGL (610860)*	c.1183C>T	p.Gln395^b^	AR	Class 1	GSD Type III	3y	Liver
Vitamins	*BTD (609019)*	c.1420G>T	p.Gly474^b^	AR	Class 2	BTD	21m	Neuro
		c.203_206dupTCCT exon 2	p.Ser70Profs^b^2	AR	Class 2	BTD	8y	Neuro, skin, hearing
Carbohydrates	*GALT (606999)*	c.707G>T	p.Ser236Ile	AR	Class 3	Galactosemia	3y	Neuro

^a^ As classified by molecular genetic laboratory.

^b^ At genetic diagnosis.

CLass 1, pathogenic; Class 2, likely pathogenic; Class 3, VUS, variant of uncertain significance; Class 6, disease-associated variant; AR, autosomal recessive; m, month; y, year; d, day; NBS, newborn screening; as, asymptomatic; BH4, tetrahydrobiopterin; TYR, tyrosinemia; MSUD, maple syrup urine disease; HMG CoA lyase, 3-Hydroxy-3-methylglutaryl-CoA lyase; 3-MGC, 3-Methylglutaconic aciduria; MMDS4, multiple mitochondrial dysfunctions syndrome-4; COXPD40, Combined oxidative phosphorylation deficiency-40; MTDPS, Mitochondrial DNA depletion syndrome; cyt c oxidase, cytochrome c oxidase; Oxphos, oxidative phosphorylation; MPS, mucopolysaccharidosis; GSD, glycogen storage disease; BTD, biotinidase deficiency.

**FIGURE 2:**
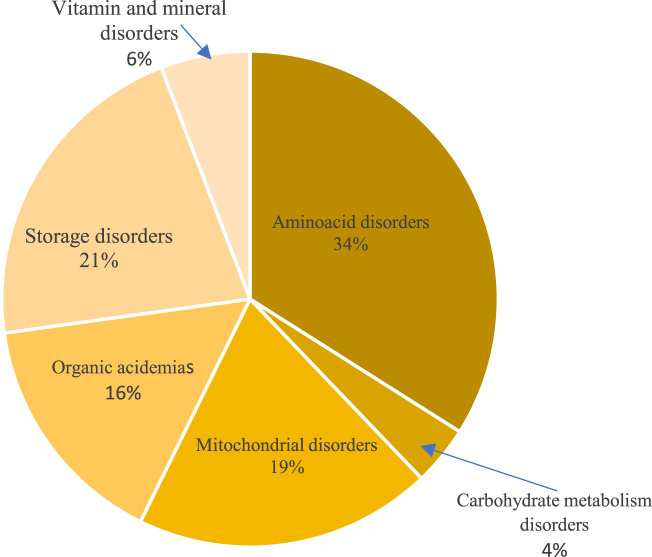
Distribution of inborn errors of metabolism categories detected by genetic testing.

Amino acid disorders were the most common identified IEM category (34%). Classical phenylketonuria was the most common aminoacid disorder detected (12%), followed by urea cycle defects (8%) with missense mutations found in half of the phenylketonuria cases and all citrullinemia and ornithine transcarbamylase deficiency patients. Storage disorders were identified in 21%, mostly mucopolysaccharidosis and glycogen storage diseases with underlying pathogenic mutations in most of these cases. Mitochondrial disorders and organic acidemias were genetically confirmed in 21% and 16%, respectively. Leigh syndrome was the most common among mitochondrial diseases with an underlying SURF1 gene pathogenic mutation in 62%. Among organic acidemias, cobalamin metabolism disorders were the most common (5%), caused by frameshift mutations in MMACHC gene, in the majority of cases. Other disorders like vitamin and mineral disorders along with galactosemia and glucose transporter deficiency reached 10% (Figure 2). All biotinidase deficiency patients exhibited likely pathogenic mutations in the BTD gene, while missense mutations were found in galactosemia patients (Supplementary Table S1).

Genetic diagnostic confirmation of IEM categories allowed the implementation of a specific medical and/or dietary management for treatable IEM, like aminoacid and organic acid disorders, mucopolysaccharidosis, glycogen storage diseases, biotinidase deficiency, Wilson disease and galactosemia. All families benefited from timely genetic counseling, even if only symptomatic treatment could be offered in some IEM case, as in mitochondrial diseases, for example. Furthermore, genetic testing of index patients allowed the detection of 16 cases in 13 families, while they were still asymptomatic or had early non-specific symptoms, leading to early medical care. Unfortunately, 23 patients out of 126 (18%) had negative genetic results (WES or single gene sequencing) despite suggestive clinical profile and biochemical investigations (Table 3). All these patients did not undergo further testing for financial reasons.

**TABLE 3:** Suspected IEM patients with negative genetic testing: Clinical and biochemical correlation.

Patient	Age	Sex	System	Biochemical tests	Suspected disease	Genetic test/gene
M1	4y	F	Skeletal	Blood enzyme assay Urine GAG	MPS type IVa	SG
M2	7y	F	Skeletal	Blood enzyme assay Urine GAG	MPS type IVa	SG
M3	8y	F	Skeletal, cardiac, liver	DBS, urine	MPS type VI	SG
M4	2y	M	Liver	Hypoglycemia, transaminitis	GSD type III	SG
M5	2m	M	Liver, muscle, neurological	Lactic acidosis, RC assay muscle	Mito complex I deficiency	SG
M6	1y	M	Liver	Neonatal screening, hypoglycemia	SCAD deficiency	SG
M7	5m	F	Neurological	Lactic acidosis, RC assay muscle	Mito complex IV	SG
M8	9y	F	Liver	AC profile, enzyme assay skin	CPT 1A	SG
M9	15y	F	Liver, muscle	AC profile, hypoketotic hypoglycemia, rhabdomyolysis	VLCAD	SG
M10	7y	M	Neurological	Enzyme assay on DBS		
M11	4y	M	Liver, spleen, neurological	Enzyme assay on DBS	Nieman- Pick A, B	P
M12	2y	M	Neurological	plasma, urine creatine	Creatine deficiency	WES trio
M13	7m	M	Liver, skeletal	Cholestasis	unsure	WES solo
M14	4y	M	Liver	Hepatic failure, LA, hyperalaninemia	Mito disease	WES solo
M15	18m	M	Neurological	LA, hyperalaninemia	Mito disease	WES trio
M16	22d	F	Neurological	LA, hyperalaninemia	Mito disease	WES trio
M17	2y	F	Neurological	Creatine, guanidinoacetic acid	GAMT deficiency	WES trio
M18	10d	F	Neurological	RC assay muscle	Mito disease	WES, mtDNA
M19	7y	F	Neurological	RC assay muscle	Mito disease	WES, mtDNA
M20	11m	M	Neurological	LA, hyperalaninemia	Mito disease	WES, mtDNA
M21	10y	M	Neurological	LA, hyperalaninemia	Mito disease	WES, mtDNA
M22	20y	F	Neurological, cardiac	LA, hyperalaninemia	Mito disease	WES, mtDNA
M23	2m	F	Liver	Hypoketotic hypoglycemia, anemia	Mito disease	WES, mtDNA

y, year; m, months; d, days; GAG, glycosaminoglycans; DBS, dried blood spots; RC, respiratory chain; AC, acylcarnitine profile; LA, lactic acidosis; MPS, mucopolysaccharidosis; GSD, glycogen storage disease; SCAD, short- chain acyl-CoA dehydrogenase deficiency; Mito, mitochondrial; CPT1A, carnitine palmitoyltransferase 1A deficiency; VLCAD, very long-chain acyl-CoA dehydrogenase deficiency; GAMT deficiency, guanidinoacetate methyltransferase deficiency; SG, single gene sequencing; P, gene panel sequencing; WES, whole exome sequencing; mtDNA, mitochondrial DNA sequencing.

### Genetic testing and diagnostic yield

Genetic testing identified mutations in 82% (103/126) of the patients and revealed disease-causing class 1 or class 2 variants in 80 probands, rendering a diagnostic yield of 63% (80/126). In the cohort of 126 probands, single gene testing was mostly requested (53%), followed by WES (36%) and gene panels (10%).

Diagnosis of IEM categories followed a similar trend over the years of the study period; Targeted gene testing, including single gene sequencing or gene panels, was predominantly ordered in 77% of patients in case of amino acid and organic acid disorders, and in 55% of suspected storage disorders, whenever biochemical diagnosis was strongly depicting a particular enzymatic deficiency. Untargeted WES ± mt DNA sequencing were mostly requested if a targeted gene test was negative or for complex cases, like in 55% of those suspected of mitochondrial disorders. WGS was done only once because of its prohibitive cost. Diagnostic yield was the highest for single gene sequencing reaching 75%, whereas WES ± mtDNA was able to confirm the diagnosis in almost half of the probands (Table 4).

**TABLE 4:** Diagnostic yield of each gene test type in a cohort of 126 patients.

Genetic test (total number)	N (126)	Positive (80)	VUS (23)	Negative (23)	Diagnostic yield 80/126 (63.4%)
Single gene	67 (53%)	50	7	10	75%
Panel	13 (10%)	8	4	1	61%
WES ± mtDNA	45 (36%)	22	11	12	49%
WGS	1 (1%)	—	1	—	—

N, number of patients; VUS, variants of unknown significance; WES, whole exome sequencing; mtDNA, mitochondrial DNA; WGS, whole genome sequencing. Positive results: class 1 or class 2 mutations.

## Discussion

NGS advances during the last decade helped identify a lot of IEM disorders known to be mostly prevalent in countries with high consanguinity rates, like Lebanon. In this study, consanguinity reached 67% with a history of affected family members identified in half of the cases. Only 7% were diagnosed by newborn screening while the majority presented when symptomatic, at various ages up to adulthood. Presenting clinical signs were non- specific, affecting mostly the neurological system, followed by other organ involvement like liver, bones, heart etc. as reported in studies of clinical IEM presentation (Agana et al., 2018). Aminoacidopathies and organic acidemias are the most frequent disorders diagnosed (Karam et al., 2013) in Lebanon, followed by lysosomal and mitochondrial diseases.

Molecular findings by NGS confirmed IEM diagnosis in 63.4% of cases, featuring 64 pathogenic or likely pathogenic variants in 80 patients, of which 67 (84%) had amenable to treatment disorders. VUS interpretation remains the main challenge for both the geneticist and the clinician. VUS, found in 22% of cases, could not be solely considered for IEM diagnosis, however, patients carrying these VUS exhibited a good genotype-phenotype correlation allowing us to confirm the diagnosis and offer a tailored management. For instance, two siblings with clinical and biochemical features of glutaric aciduria type I were homozygous for a familial missense VUS mutation, c.349G>A (p.Gly117Arg) in the *GCDH* gene, that was considered possibly linked to the disease as both parents were found heterozygote carriers for this mutation.

For patients who had negative targeted sequencing despite highly suggestive clinical and biochemical features, WES was requested but not performed for financial reasons. In the remaining, even when WES was performed, the diagnosis remained unclear in half of the patients. This highlights the need of re-interpretation of WES or even WGS later in time, integrating clinical data combined to genetic databases, as this might change the status of a VUS, likely pathogenic, likely benign, or benign variants (Gibson et al., 2018; Ji et al., 2021).

These findings highlight the importance of genetic testing for IEM and counselling in high- risk populations, however reports focusing on genetic diagnosis of IEM are still scarce. A study from Qatar reported WES utility in Mendelian disorder with few IEM cases included (Yavarna et al., 2015), while in another from Saudi Arabia (Alfares et al., 2017), WES diagnostic yield reached 49% in a large cohort of 454 patients including 59 IEM patients. Recently, a multicenter study of 213 cases from Lebanon about NGS utility in pediatric practice (Megarbane., 2018), reported mostly on neurogenetic disorders with few IEM cases. In parallel, a European study (Tarailo-Graovac et al., 2016) reported a WES diagnostic yield of 68% in 41 patients with unexplained metabolic phenotype.

The diagnostic yield of genetic testing for IEM reached 63% in our sample size of 126 probands, this may be explained by the high frequency of homozygous mutations precluding for autosomal recessive disorders in Lebanon (Nakouzi et al., 2015). Targeted gene testing was more rewarding in attaining a rapid diagnosis, with a diagnostic yield of 61%–75%, especially for treatable IEM like amino acid and organic acid disorders and some storage disorders. In parallel, a study by Yubero et al. (2016) reported a diagnostic yield of a targeted genetic panel of 78% in a group of patients who had clinical and biochemical features suggestive of IEM.

Despite the popularity and easy availability of untargeted testing including WES and even WGS, cost remains one of the limiting factors in applying these techniques. Some centers in China are currently even proposing IEM diagnosis by WES neonatal screening combined to Tandem Mass Spectrometry (Yang et al., 2019) which could be an attractive option; however, in countries like Lebanon where expanded newborn screening is not even covered yet by any third party, this remains difficult to apply. Therefore, arises the need for a judicious choice of genetic investigations for IEM diagnosis, since all genetic testing are not covered by any insurance. A high index of clinical suspicion can orient the biochemical investigations in such disorders. Biochemical genetics investigations may be used to depict the biochemical profile of aminoacidopathies and organic acidemias (Karam et al., 2013), whereas enzymatic assays on dried blood spots point, in most of the cases, to the culprit enzyme in suspected lysosomal disorders, biotinidase deficiency or galactosemia, for example. In this study, single gene testing or a multi-gene panel were able to confirm the diagnosis in 77% of amino acid and organic acid disorders, and in 55% of lysosomal storage disorders. Similarly, a recent study by Barbosa-Gouveia et al., 2021, prospectively analyzed the diagnostic yield of four designed different multi-gene panels based on the presenting symptoms of 311 patients. A high diagnostic yield was demonstrated in case of intermediary metabolism defects (61.86%), while it reached 17% for mitochondrial diseases.

Choosing targeted genetic testing can alleviate the cost of resorting to WES or WGS, especially in countries with limited resources. Patients with complex phenotypes and multi-organ involvement, like in mitochondrial diseases, could be candidates for WES as a first-tier genetic testing, if financially possible. In this study, WES diagnostic yield was 49%. Almost half of the patients suspected of mitochondrial diseases were investigated by WES as a first-tier testing, thus avoiding invasive muscle or skin biopsy, however, a negative result in such cases would warrant further expenses, whether for genetic testing on an affected tissue or for proceeding to WGS ± mtDNA to confirm the diagnosis. Our findings parallel those of a study by Kose et al. (2021), advocating for the use of WES as the method of choice for mitochondrial diseases diagnosis.

Single gene and well updated, specially designed panel sequencing can offer rapid and affordable diagnostic solutions whenever amino acid, organic acid, or storage disorders are suspected. WES can identify complex IEM cases like mitochondrial disorders, peroxisomal or even other complex defects. The results presented here encourage the incorporation of NGS as a primary investigational tool for the diagnosis of all IEM categories in clinical practice, although Sanger can still be adopted for single gene sequencing. Clinically skilled physicians and focused biochemical testing play a major role in orienting NGS diagnostic conclusions.

Finally, offering WES as a first-tier test may deter both the physician and the patient as it may seem too expensive, however, the expenses incurred by patients for a metabolic work-up added to that of basic blood and urine studies or even tissue biopsies, may be equivalent to, if not more than that of WES or WGS cost. Further studies exploring the cost-effectiveness of adopting NGS as first-tier testing in IEM diagnosis are needed.

## Data Availability

The datasets presented in this study can be found in online repositories. The names of the repository/repositories and accession number(s) can be found in the article/Supplementary Material.
