# Supplementary material for: Challenges of genetic diagnosis of inborn errors of metabolism in a major tertiary care center in Lebanon
Source: Front Genet. 2022 Nov 18;13:1029947. doi: 10.3389/fgene.2022.1029947 (PMC9715967; doi:10.3389/fgene.2022.1029947)
Supplement: Supplementary file 1 [file Table1.DOCX]

**Table 1- Supplementary**: Pathogenic and likely pathogenic variants identified in 80 IEM patients

Legend: ^1^As classified by molecular genetic laboratory, ^2;^ at genetic diagnosis, ^ unreported, AR: Autosomal Recessive, XL: X-linked, AD: Autosomal Dominant, class 1: pathogenic, class 2: likely pathogenic, class 3: variant of uncertain significance, class 4: likely benign, class 5: benign, class 6: disease-associated variant, def: deficiency, NBS: newborn screening, as: asymptomatic, HSM: Hepatosplenomegaly, PKU: phenylketonuria, BH4: Tetrahydrobiopterin, TYR: Tyrosinemia , MSUD: Maple syrup urine disorders, HHH: hyperornithinemia-hyperammonemia-homocitrullinuria OTC: ornithine transcarbamylase deficiency, NKH:NonKetotic Hyperglycemia, MMA: Methylmalonic academia, Cbl : cobalamin , HMG CoA Lyase: 3-Hydroxy-3-methylglutaryl-CoA lyase, PA: Propionic academia, IVA: Isovaleric academia, MMDS4: Multiple mitochondrial dysfunctions syndrome type 4, RSL1- RC: RSL1-Respiratory chain, MTDPS: Mitochondrial DNA depletion syndrome, MCAD: Medium-chain acyl-CoA dehydrogenase, GM1:gangliosidosis type I, MPS: Mucopolysaccharidosis, GSD: Glycogen storage

disease, BTD: Biotinidase deficiency.

| **Category** | | **Gene** | **Molecular Genetics** | | **Protein effect** | | **Mutation type** | | **inheritance** | | **Variant^1^** | | **Disease** | | **Age^2^** | **System involved** |
| --- | --- | --- | --- | --- | --- | --- | --- | --- | --- | --- | --- | --- | --- | --- | --- | --- |
| **Amino acid**  **disorders** | | *PAH* | IVS7+1G>T c.842+1G>T | | NA | | Splice donor | | AR | | class 1 | | PKU | | 1y | neuro |
|  |  | *PAH* | c.143T>C | | p.leu48ser | | Missense | | AR | | class 1 | | PKU | | 1y4m | neuro |
|  |  | *PAH* | c.1066-11G>A | | NA | | Intron | | AR | | class 1 | | PKU | | 4y | neuro |
|  |  | *PAH* | c.1066-11G>A | | NA | | Intron | | AR | | class 1 | | PKU | | 6y | neuro |
|  |  | *PAH* | IVS10-11G>A | | NA | | Intron | | AR | | class 1 | | PKU | | 3y | neuro |
|  |  | *PAH* | IVS10-11G>A c.1066-11G>A | | NA | | Intron | | AR | | class 1 | | PKU | | 2y | neuro |
|  |  | *PAH* | c.473G>A | | p. Arg158Gln | | Missense | | AR | | class 1 | | PKU | | 3y | neuro |
|  |  | *PAH* | c.473G>A | | p. Arg158Gln | | Missense | | AR | | class 1 | | PKU | | 2m | NBS- as |
|  |  | *PAH* | c.1222C>T; c.1066-11G>A (IVS10-11G>A) | | p. Arg408Trp | | Missense,  Intron | | AR | | class 1 | | PKU | | 1m | NBS-as |
|  |  | *PAH* | c.727C>T; T380M; c.1139C>T | | p. Arg243Ter; p. Thr380Met | | -Nonsense  - Missense | | AR | | Class 1 | | PKU mild | | 3m | NBS-as |
|  |  | *PTS* | c.317C>T | | p. Thr106Met | | Missense | | AR | | class 2 | | BH4 type A | | 2m | neuro |
|  |  | *QDPR* | c.197A>G ^ | | p. Gln66Arg^ | | Unknown | | AR | | Class 1 | | BH4 type C | | 22m | neuro |
|  |  | *FAH* | c.1210G>A | | p. GLY404Ser | | Missense | | AR | | Class 1 | | TYR-I | | 3y | liver, kidney |
|  |  | *FAH* | c.1062+5G>A | | NA | | Intron | | AR | | Class 1 | | TYR-I | | 2y | liver |
|  |  | *FAH* | c.554-1G>T;  c.692T>C;  c.1054A>G ^ | | NA  p. Met231Thr  p. Ser352Gly ^ | | Splice acceptor  Missense  Unknown | | AR | | class 1/3/3 | | TYR-I | | 2y | Liver kidney |
|  |  | *OCA2* | c.2036G>A ^ | | p. Trp679* | | Unknown | | AR | | Class 2 | | Albinism -II | | 5m | skin |
|  |  | *DBT* | c.224G>A | | p. Gly75G | | Unknown | | AR | | Class 1 | | MSUD | | 4y | neuro |
|  |  | *DBT* | c.224G>A | | p. Gly75G | | Unknown | | AR | | Class 1 | | MSUD | | prenatal | as |
|  |  | *BCKDHA* | c.890G>A | | p. Arg297His | | Missense | | AR | | Class 1 | | MSUD | | 6y | Neuro,liver |
|  |  | *ASS1* | c.787G>A | | p. Val263Met | | Missense | |  | | Class 1 | | Citrullinemia | | 10y | neuro/liver |
|  |  | *ASS1* | c.787G>A | | p. Val263Met | | Missense | | AR | | Class 1 | | Citrullinemia | | 14y | neuro |
|  |  | *ASS1* | c.910C>T | | p. Arg304Trp | | Missense | | AR | | Class 1 | | Citrullinemia | | 8y | neuro/liver |
|  |  | *ASS1* | c.535T>C | | p. Trp179Arg | | Missense | | AR | | Class 1 | | Citrullinemia | | 4m | NBS/as |
|  |  | *ASS1* | c.910C>T | | p. Arg304Trp | | Missense | | AR | | Class 1 | | Citrullinemia | | 1y | neuro |
|  |  | *SLC25A15* | c.562_564del | | p. Phe188del | | Inframe deletion | | AR | | Class 1 | | HHH | | 12y | neuro/liver |
|  |  | *SLC25A15* | c.562_564del | | p. Phe188del | | Inframe deletion | | AR | | Class 1 | | HHH | | 17y | neuro/liver |
|  |  | *OTC* | c.622G>A | | p. Ala208Thr | | Missense | | XL | | Class 1 | | OTC | | 42y | as |
|  |  | *OTC* | c.622G>A | | p. Ala208Thr | | Missense | | XL | | Class 1 | | OTC | | 30y | neuro/liver |
|  |  | *OTC* | c.119G>A | | p. Arg40His | | Missense | | XL | | Class 1 | | OTC | | 26y | neuro/liver |
|  |  | *AMT* | c.452_466del | | p. Lys151_Leu155del | | Inframe deletion,intron | | AR | | Class 1 | | NKH | | 1y | Neuro |
|  | | | | | | | | | | | | | | | | |
| **Organic acid**  **disorders** | *MMUT* | | | c.322C>T | | p. Arg108Cys | Missense (Exon 2) | AR | | class 1 | | MMA | | | 5y | liver |
|  | *MMUT* | | | c.322C>T | | p. Arg108Cys | Missense (Exon 3) | AR | | class 1 | | MMA | | | 2y | liver/neuro |
|  | *MMUT* | | | c.655A>T; c.1871A>G | | p. Asn219Tyr; p. Gln624Ar | Missense | AR | | class 1 | | MMA | | | 16y | Neuro/liver |
|  | *MMACHC* | | | c.271dup | | p. Arg91Lysfs | Frameshift | AR | | class 1 | | Cbl c | | | 5y | neuro |
|  | *MMACHC* | | | c.217C>T; c.271dup | | p. Arg73Ter; p. Arg91Lysfs | Nonsense; Frameshift | AR | | class 1 | | Cbl c | | | 11m | neuro |
|  | *MMACHC* | | | c.547_548del | | p. Val183fs | Frameshift | AR | | class 1 | | Cbl c | | | 19y | neuro |
|  | *MMAB* | | | c.568C>G | | p. Arg190Gly | Missense | AR | | class2 | | Cbl b | | | 18m | neuro |
|  | *ASPA* | | | c.497C>T ^ | | p. Thr166IIe ^ | Unknown | AR | | Class 6^ | | Canavan | | | 2y | neuro |
|  | *ASPA* | | | c.914C>A | | p. Ala305Glu | Missense-Intron | AR | | class 1 | | Canavan | | | 8y5m | neuro |
|  | *HMGCL* | | | c.918dup ^ | | p. Cys307Leufs*9^ | Unknown | AR | | class 2 | | HMG CoA lyase | | | 1m | neuro |
|  | *PCCA* | | | c.1209+3A>G | | NA | Intron | AR | | class 1 | | PA | | | 2y | Neuro  /liver |
|  | *IVD* | | | Large deletion exons 1, 2, 3 | | NA | deletion | AR | | class2 | | IVA | | | 14y | liver |
|  | | | | | | | | | | | | | | | | |
| **Mitochondrial disorders** | ISCA2 | | | c.37_39del^ ;  c.190_192del^ | | p. Thr13del^; p. Thr64del^ | unknown | AR | | class 2 | | MMDS4 | | 10m | | neuro |
|  | QRSL1 | | | c.173G>T ^ | | p. Arg58IIe ^ | unknown | AR | | class2 | | RSL1- RC | | 1m | | cardiac |
|  | FBXL4 | | | c.1303C>T | | p. Arg435Ter | Nonsense | AR | | class2 | | MTDPS13 | | 4m | | neuro |
|  | PET100 | | | c.3G>C | | p. Met1Ile | missense | AR | | class 1 | | Complex IV | | 8m | | neuro |
|  | SURF1 | | | c.870delT^ | | p. F290fs ^ | Unknown | AR | | class 1 | | Leigh | | 1y9m | | neuro |
|  | SURF1 | | | c.370G>A ^ | | p. Gly124Arg ^ | Unknown | AR | | class 1 | | Leigh | | 1y | | neuro |
|  | SURF1 | | | c.370G>A ^ | | p. Gly124Arg ^ | Unknown | AR | | class 1 | | Leigh | | 9y | | neuro |
|  | MPV17 | | | c.148C>T; c.284dup ^ | | p. Arg50Trp; p. Phe96Leufs*17^ | - Missense  -Unknown | AR | | class2 | | MTDPS | | 1y | | liver |
|  | SURF1 | | | c.370G>A ^ | | p. Gly124Arg ^ | Unknown | AR | | class 1 | | Leigh | | prenatal | | as |
|  | SURF1 | | | c.370G>A^ | | p. Gly124Arg ^ | Unknown | AR | | class 1 | | Leigh | | 2y | | neuro |
|  | FBXL4 | | | c.605 C>G ^ | | p. Thr202 Arg^ | Unknown | AR | | class2 | | MTDPS | | 13y | | neuro |
|  | ACADM | | | c.985A>G | | p. Lys329Glu | Missense | AR | | class2 | | MCAD | | 1m | | Nbs/as |
|  | ACADM | | | c.985A>G | | p. Lys329Glu | Missense | AR | | class2 | | MCAD | | 10y | | as |
|  | | | | | | | | | | | | | | | | |
| **Storage disorders** | NPC1 | | | c.2098G>A | | p. Asp 700Asn | Missense | AR | | Class 1 | | Niemann Pick Type C1 | | 16y | | neuro |
|  | NPC1 | | | c.2098G>A | | p. Asp 700Asn | Missense | AR | | Class 1 | | Niemann Pick Type C1 | | 10y | | neuro |
|  | SMPD1 | | | c.923del ∧ | | p. Phe308Serfs*77 ∧ | unknown | AR | | Class 1 | | Niemann-Pick type A/B | | 6m | | neuro |
|  | GLB1 | | | c.320G>A ∧ | | p. Arg107His ∧ | unknown | AR | | Class 1 | | GM1-gangliosidosis | | 1y | | neuro |
|  | GAA | | | c.1209delC | | p. Asn403Lysfs*37 | Frameshift | AR | | Class 1 | | Pompe | | 1y6m | | neuro |
|  | IDUA | | | c.1882C>T | | p. Arg628Ter | nonsense | AR | | Class 1 | | MPS type I | | 5y | | HSM, neuro, skeletal |
|  | ARSB | | | C.962T>C | | p. Leu321Pro | missense | AR | | Class 1 | | MPS type VI | | 1y7m | | skeletal |
|  | ARSB | | | c.691-13A>G | | NA | unknown | AR | | Class 6 | | MPS type VI | | 14y | | Skeletal, cardiac |
|  | ARSB | | | c.691-13A>G | | NA | unknown | AR | | Class 6 | | MPS type VI | | 8y | | Skeletal, cardiac |
|  | SGSH | | | C.1298G>A | | p. Arg433Gln | missense | AR | | Class 1 | | MPS type IIIA | | 2y | | neuro/hearing |
|  | IDS | | | c.257C>T | | p. Pro86Leu | missense | XLR | | Class 1 | | MPS type II | | 8y | | neuro/skeletal |
|  | GALNS | | | c.901G>T | | p. Gly301Cys | missense | AR | | Class 1 | | MPS type VI | | 2y | | neuro/skeletal |
|  | IDUA | | | c.1882C>T | | p. Arg628Ter | nonsense | AR | | Class 1 | | MPS I Hurler | | 4y | | Neuro/skeletal |
|  | G6PC | | | c.247C>T | | p. Arg83Cys | missense | AR | | Class 1 | | GSD Ia | | 1y2m | | liver |
|  | G6PC | | | c.247C>T p. Arg83Cys | | p. Arg83Cys | missense | AR | | Class 1 | | GSD 1a | | 5m | | liver |
|  | G6PC | | | c.497T>G | | p. Val166Gly | missense | AR | | Class 1 | | GSD 1a | | 2y | | liver |
|  | AGL | | | c.1183C>T | | p. Gln395* | unknown | AR | | Class 1 | | GSD Type III | | 3y | | liver |
|  | GBE1 | | | c.986A>G  c.1322A>G | | p. Tyr329Cys; p. Asp441Gly | missense | AR | | Class 1/VUS | | GSD type IV | | 1y10m | | liver |
|  | | | | | | | | | | | | | | | | |
| **Vitamin and mineral disorders** | BTD | | | c.1420G>T | | p. Gly474* | Unknown | AR | | class2 | | BTD | | 21m | | neuro |
|  | BTD | | | c.203_206dupTCCT exon 2 | | p. Ser70Profs*2 | Unknown | AR | | class2 | | BTD | | 8y | | Neuro, skin, hearing |
|  | BTD | | | c.203_206dupTCCT exon 2 | | p. Ser70Profs*2 | Unknown | AR | | class2 | | BTD | | 11y | | Neuro, skin |
|  | ATP7B | | | c.1707+2dup exon 4 | | NA | Splice Donor | AR | | class 1 | | Wilson Disease | | **7y** | | liver |
|  | SLC52A2 | | | c.916G>A | | p. Gly306Arg | missense | AR | | Class 1 | | Riboflavin | | 5y | | neuro |
|  | | | | | | | | | | | | | | | | |
| **Carbohydrate metabolism disorders** | GALT | | | c.983G>A | | p. Arg328His | missense | AR | | class 1 | | Galactosemia | | 2y 7m | | ophthalmic |
|  | SLC2A1 | | | c.1279-2A>G | | NA | Splice Acceptor | AD/AR | | class2 | | Glucose transporter deficiency type 1 | | 15m | | neuro |
